# Gender Disparities in Nephrology Trials: A Meta-Analysis of Enrollment Trends between 2000 and 2021

**DOI:** 10.34067/KID.0000000000000281

**Published:** 2023-10-27

**Authors:** Qandeel H. Soomro, Angela McCarthy, Amalya M. Charytan, Colin Keane, Dalila Varela, Javaughn Ways, Giana Ramos, Joey Nicholson, David M. Charytan

**Affiliations:** 1 Division of Nephrology, NYU Grossman School of Medicine, NYU Langone Health, New York, New York; 2 NYU Health Sciences Library, NYU Grossman School of Medicine, NYU Langone Health, New York, New York

**Keywords:** AKI, CKD, dialysis, ESKD, gender difference, GN

## Abstract

**Key Points:**

- Women are under-represented in high-impact nephrology trials.
- Trends remain consistent over the past 20 years and on the basis of target condition.
- Addressing the imbalanced enrollment of women in trials could improve disparities in care and outcomes of kidney disease.

**Background:**

Gender disparities in the incidence and complications of kidney diseases are well described. However, analysis to elucidate gender disparities in enrollment in nephrology randomized clinical trials (RCTs) has not been performed.

**Methods:**

We performed a systematic review and meta-analysis of high-impact nephrology RCTs published between 2000 and 2021. We included RCTs enrolling participants aged 18 years and older in the following categories: (*1*) CKD, (*2*) AKI, (*3*) GN, (*4*) maintenance dialysis, and (*5*) kidney transplantation. We summarized trial characteristics according to reporting and enrollment of participants, enrollment site, publication year, trial category, and intervention type. Outcomes of interest include the proportion of enrolled male and female participants overall and according to trial category. In addition, we compared enrollment trends in the United States and globally to estimates of kidney disease prevalence.

**Results:**

Most qualifying trials (373/380, 98%) reported the distribution of male and female participants. Enrollment was imbalanced overall with male participants accounting for 62% (*n*=215,850) of the enrolled participants and female participants for just 38% (*n*=133,082). Male participants formed most of trial cohorts in AKI (65%), CKD (62%), dialysis (55%), and transplant trials (65%), whereas women were majority enrollees in GN trials (61%). CKD trials under-represented women in both US trials and worldwide.

**Conclusions:**

Women are under-represented in high-impact nephrology trials with the exception of GN trials. This imbalance may contribute to disparities in outcomes and gaps in the care of women with kidney disease.

## Graphical abstract

**Figure:**
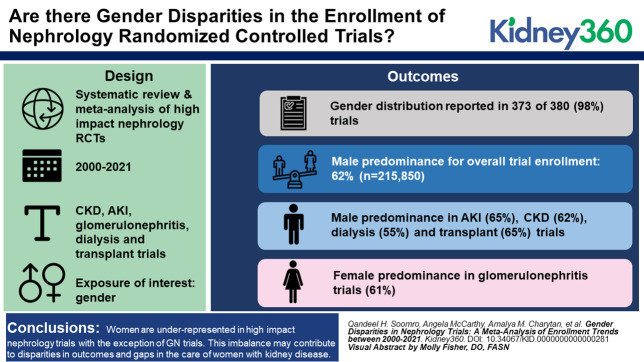

## Introduction

Adequate representation of women in clinical trials is crucial to understanding the relationship between disease progression and gender and to guide the development of new treatments. Nonetheless, there has been gross under-enrollment of women in general clinical trials for decades,^1,2^ perhaps stemming from a historical 1977 US Food and Drug Administration (FDA) recommendation for the exclusion of women of childbearing potential from all phase I and early phase II clinical research.^3^ Although the National Institutes of Health (NIH) Revitalization Act of 1993 attempted to rectify this information loss by mandating adequate inclusion of women in NIH-sponsored clinical trials, the proportion of women enrolled in clinical trials overall remains suboptimal.^3–5^

Representative enrollment of women in kidney disease trials may be particularly important for ensuring that standard therapies are effective for patients of all sexes and genders and in reducing disparities in care. It is important to consider this within the context of the gender disparities that exist in the global burden of CKD. For example, in a 2016 study, there was a markedly lower prevalence of CKD in men than women, with a male to female ratio of 0.81; however, the incidence of progression to kidney replacement therapy and mortality was lower.^6^ Several hypotheses have been proposed to explain sex-related differences in kidney disease morbidity and mortality, including hormonal influences on inflammatory and immunological responses, resistance to oxidative stress, and differences in fat storage.^7,8^ In particular, reproductive hormones play a key role in “sexual dimorphism” (gender and sex differences) in a variety of organs, including the kidneys, where they regulate various structural and functional aspects of kidney function and response to pharmacologic agents.^7,9^ However, to the best of our knowledge, the enrollment of women in nephrology trials in high-impact journals over the past 20 years has not been systematically evaluated. To understand sex-specific trends in nephrology trial enrollment, we performed a systematic review and meta-analysis of the reporting and enrollment of male and female participants in nephrology clinical trials.

## Methods

### Study Selection

We used a recently assembled database including high-impact nephrology trials. Study selection and search strategy for this dataset have been previously reported in detail.^10^ In brief, we identified randomized clinical trials (RCTs) published between 2000 and 2021 in ten high-impact journals, with the objective of identifying trials most likely to influence standards of care. We included RCTs enrolling participants aged 18 years and older with the following target population: (*1*) CKD, (*2*) AKI, (*3*) GN, (*4*) maintenance dialysis (hemodialysis and peritoneal dialysis), and (*5*) kidney transplantation. Trials were required to have hard clinical outcome measures, including mortality/survival, CKD progression, cardiovascular events, relapse and remission for GN trials, and renal recovery, response to therapy, and rejection for transplant trials. For AKI trials, additional outcomes such as intensive care unit, ventilation or dialysis free days, and intensive care unit discharge were allowed. Trials with <50 participants and those not meeting the adult age cutoff were excluded.

### Search Strategy

A medical librarian (J. Nicholson) designed the literature search criterion in collaboration with the other authors. Search terms are listed in Supplementary Table 1. We searched PubMed/MEDLINE on January 27, 2022, with search terms designed to capture RCTs for the five kidney disease–related conditions described above. To focus on trials likely to influence standards of care, we limited our search to studies published in ten high-impact journals with high impact factors: *American Journal of Kidney Diseases*, *American Journal of Transplantation*, *The British Medical Journal*, *CJASN*, *The Journal of American Medical Association*, *JASN*, *Kidney International*, *The Lancet*, *Nephrology Dialysis and Transplantation*, and *The New England Journal of Medicine*, between January 1, 2000, and December 31, 2021.

### Screening and Data Collection

Study screening was performed independently by at least two authors using data collection and processing software (Covidence, Veritas Health Innovation, Melbourne, Australia). The initial stage screened studies on the basis of titles and abstracts only. Studies were then identified for eligibility using full-text review of published articles. Disagreements were resolved by consensus and finalized by the first and last authors. Data were abstracted for key variables by two authors (Q.H. Soomro and A. McCarthy), including demographics, study design and target condition, sample size, study geographical region/location (defined below), and outcomes of interest. Data were abstracted from original papers, supplementary material, protocol papers, and the ClinicalTrials.gov website. As needed, authors were contacted for clarification regarding demographics, funding agency, and trial sites.

Interventions were categorized as drug, device, dialysis prescription, procedure or surgery, care delivery, exercise/mindfulness, or other. Funding sources were categorized as NIH, non-NIH government (*i.e.*, non-US governmental funding or funding by the US FDA or Department of Veteran Affairs), industry, foundation, or other. Trial enrollment was also categorized according to region as the United States only, international (outside of the United States only), Europe, Asia, and global (both US and international enrollment). Publication year was categorized as 2000–2006, 2007–2011, 2012–2016, or 2017–2021. Trial quality was analyzed using the Cochrane tool.^11^

### Statistical Analysis

Trial characteristics are summarized according to reporting and enrollment of participants, enrollment sites, publication year, target condition, and intervention type. Outcomes of interest included the proportion of enrolled male and female participants according to target condition categories. Additional analyses included proportions across enrollment site and publication date categories. Descriptive statistics are reported as *n* (%), mean±SD, or median (interquartile range).

Meta-analytic estimates of the proportion of participants were performed using the *metaprop* command in STATA.^12^ Pooled effect size estimates were calculated using random effects models with restricted maximum likelihood as the heterogeneity estimator. Confidence intervals for the binomial proportion were calculated using exact or Clopper–Pearson methods.^12^

The primary category of interest was the target medical condition (CKD, AKI, GN, maintenance dialysis, and kidney transplantation). We additionally performed *a priori* planned subgroup analyses according to publication date, enrollment site, and intervention using the random effects model to further explore heterogeneity. Finally, we compared global and US trial enrollment with estimates of global sex-specific population estimates using the United Nations world population estimates,^13^ US and global sex-specific CKD prevalence estimates using data from the Global Burden of Disease study^6^ and the National Health and Nutrition Examination Survery data,^14^ and ESKD and transplant data from the United States Renal Data System.^15^ Statistical analysis was performed using STATA version 17 (StataCorp, College Station, TX) and Microsoft excel. *P* < 0.05 was considered significant.

## Results

### Overall Results

As reported previously,^10^ the search strategy identified 4494 articles. Of these, 1200 were included in the full-text screening, and 380 randomized controlled trials met all inclusion criteria, 86 (23%) of included trials were CKD, 69 (18%) were AKI, 56 (15%) were ESKD on dialysis, *etc.* Despite publication in high-impact journals, there were numerous deficiencies in reporting quality (Supplementary Table 2). Most trials that reported the distribution of male and female participants was 373 of 380 (98%); one trial reported that most participants were men without providing an actual count. Enrollment was imbalanced overall with male participants accounting for 62% (*n*=215,850) of the enrolled participants and female participants for 38% (*n*=133,082), with a male to female ratio of 1.62. Randomization was stratified on the basis of sex in 23 of 373 trials (6.1%), and subgroup analysis on the basis of sex was performed in the primary outcome articles in 66 of 373 trials (17.6%).

### Gender Distribution According to Target Condition and Additional Trial Characteristics

**Figure 1:**
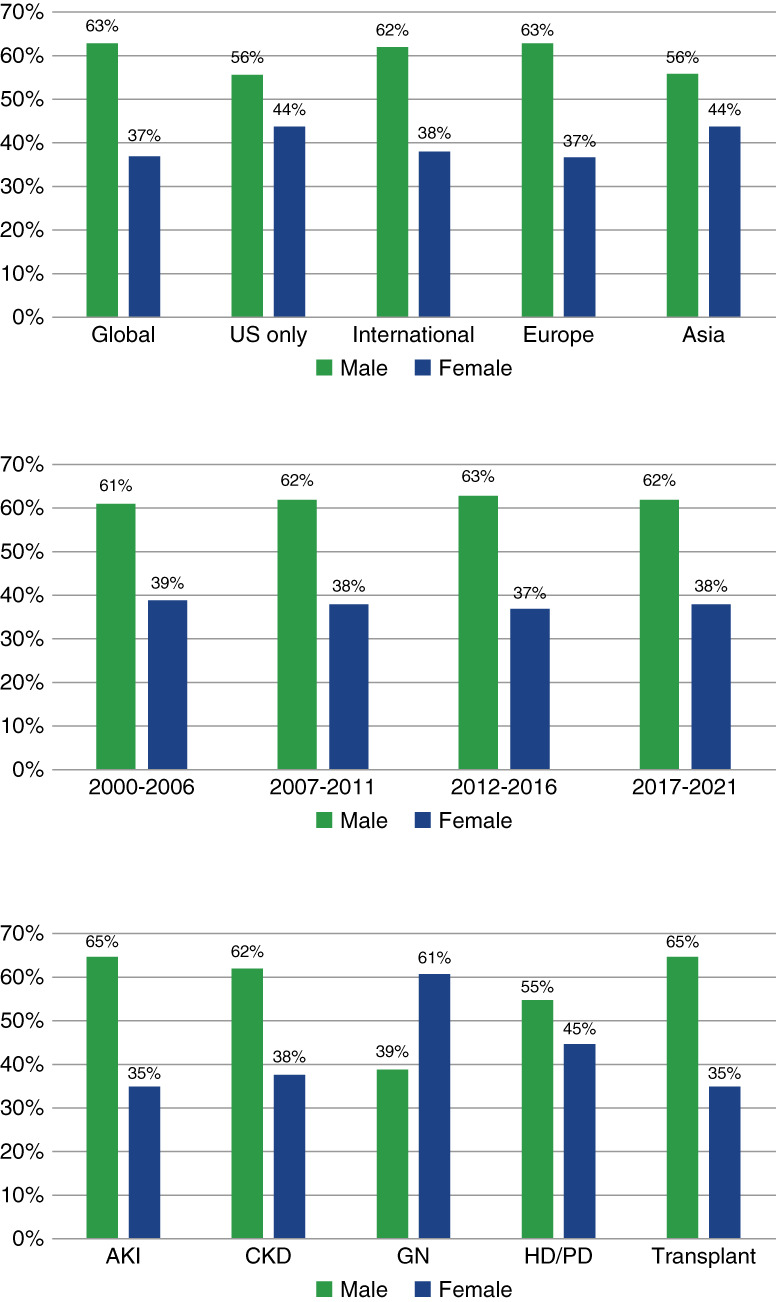
**Distribution of male and female participants in kidney disease trials according to location, publication year, and trial category.** International=non-US trial. HD, hemodialysis; PD, peritoneal dialysis.

Male participants accounted for most of the enrolled participants (61%–65%) in all trial categories with the exception of GN trials where female participants accounted for 61% of enrollees (Figure 1 and Table 1). Overall disparities in enrollment were particularly notable for AKI (65% male participants), CKD (62% male participants), and transplant trials (65% male participants) with less dramatic disparities in dialysis trials (55% male participants). There were marked differences in enrollment according to the region of trial conduct. Male participants accounted for 63% of trial populations in global, 62% in international (outside of the United States only), and 63% in European trials, but for just 56% in US and Asian trials. Overall, enrollment trends did not differ according to funding type and were qualitatively unchanged between 2000 and 2021 with male participants accounting for >60% of all trial participants in each period. As shown in Table 2, there were not trends for differential enrollment according to enrollment period and target condition. Men accounted for 67% of AKI, 61% of CKD, 35% of GN, 52% of dialysis, and 64% of trial population in 2000–2006 and 63%, 63%, 38%, 53%, and 65% of participants in 2017–2021, respectively. Interestingly, trials with procedure/surgery as an intervention enrolled relatively higher proportions of male participants (72%) compared with other categories, whereas male (54%) and female (46%) enrollment were more similarly distributed in device trials.

**Table 1:** Overall characteristics of trials

Characteristic	Trials *N* (%)^a^ Total=380	Male *N* (%) 374 (98%) No. Men Enrolled=215,850	Female *N* (%) 373 (98%) No. of Women Enrolled=133,082
**Enrollment site**			
Global	82 (22)	110,355 (63)	63,383 (37)
United States only	61 (16)	24,266 (56)	19,393 (44)
International (non-US)	41 (11)	25,325 (62)	15,677 (38)
Europe	154 (40)	49,109 (63)	29,324 (37)
Asia	41 (11)	6795 (56)	5305 (44)
**Trial category**			
AKI	69 (18)	60,029 (65)	32,757 (35)
CKD	86 (23)	92,205 (62)	55,865 (38)
GN^b^	25 (6)	1421 (39)	2266 (61)
HD/PD	56 (15)	29,157 (55)	24,024 (45)
Transplant	144 (38)	33,038 (65)	18,170 (35)
**Publication year**			
2000–2006	94 (25)	18,450 (61)	11,947 (39)
2007–2011	78 (20)	30,171 (62)	18,451 (38)
2012–2016	95 (25)	54,828 (63)	32,438 (37)
2017–2021	113 (30)	112,401 (62)	70,246 (38)
**Funding source**			
NIH	31 (8)	19,597 (60)	15,444 (40)
Non-NIH governmental	56 (15)	49,687 (67)	24,016 (33)
Industry	183 (48)	127,767 (62)	78,204 (38)
Foundation	58 (15)	28,928 (61)	18,392 (39)
Other^c^	82 (22)	20,310 (64)	11,327 (36)
**Intervention**			
Care delivery	36 (9)	39,506 (57)	29,213 (43)
Device	4 (1)	466 (54)	399 (46)
Dialysis	34 (9)	10,652 (59)	7505 (41)
Drug	281 (74)	153,552 (62)	92,678 (38)
Procedure/surgery	9 (2)	5611 (72)	2144 (28)
Exercise/meditation	2 (0.5)	280 (61)	179 (39)
Other	8 (2)	5783 (86)	964 (14)

Global=US+international trials. HD, hemodialysis; PD, peritoneal dialysis; NIH, National Institutes of Health.

a Column percentages.

b Total GN trials 25, all seven lupus nephritis trials had more women than men enrolled, and 3 of 9 IgA trials conducted in Asia had more women than men enrolled.

c Other=investigator/university funded. Some studies had more than one funding source.

**Table 2:** Enrollment by trial type and year

Target Condition	Trials *N* (%) Total=380	Male *N* (%) 374 (98%) No. of Men Enrolled=215,850	Female *N* (%) 373 (98%) No. of Women Enrolled=133,082
**AKI**			
2000–2006	15 (4)	2051 (67)	1033 (33)
2007–2011	9 (2)	2616 (64)	1441 (36)
2012–2016	20 (5)	15,466 (69)	6986 (31)
2017–2021	25 (7)	39,896 (63)	23,297 (37)
**CKD**			
2000–2006	15 (4)	4452 (61)	2904 (39)
2007–2011	22 (6)	16,114 (62)	9820 (38)
2012–2016	23 (6)	25,967 (61)	16,811 (39)
2017–2021	26 (7)	45,672 (63)	26,330 (37)
**GN**			
2000–2006	7 (2)	203 (35)	377 (65)
2007–2011	3 (1)	292 (41)	422 (59)
2012–2016	2 (0.5)	64 (52)	58 (48)
2017–2021	13 (3)	862 (38)	1409 (62)
**Dialysis**			
2000–2006	14 94)	3093 (52)	2832 (48)
2007–2011	11 (3)	4311 (59)	3052 (41)
2012–2016	15 (4)	7514 (59)	5321 (41)
2017–2021	16 (4)	14,329 (53)	12,819 (47)
**Kidney transplant**			
2000–2006	43 (11)	8651 (64)	4801 (36)
2007–2011	34 (9)	7093 (65)	3828 (35)
2012–2016	35 (9)	5817 (64)	3262 (36)
2017–2021	32 (8)	11,477 (65)	6279 (35)

### Meta-Analytic Estimates

All trials reporting enrollment information on sex of the participants were included in a meta-analysis to better estimate proportional enrollment after accounting for the number of trials and trial size. As shown in Table 3, overall and subgroup estimates were qualitatively similar in the crude and meta-analytic analyses. Overall pooled results were similar to the raw proportions with male enrollment estimated at 62% (95% CI, 60% to 64%) overall and for 67%, 61%, 48%, 59%, and 65% of AKI, CKD, GN, dialysis, and kidney transplant trials, respectively. In the weighted estimates, enrollment rates were stable across time, with only modest variation according to type of intervention. Trial disease category (*P* < 0.001) and region of enrollment (*P* = 0.02) were significantly associated with the proportion of women enrolled. Results of meta-regression analyses were similar with trial category significantly associated with the proportion of women enrolled (*P* = 0.01). By contrast, enrollment proportions were not associated with publication year, enrollment site, and intervention type (Table 4).

**Table 3:** Meta-analytic estimates of proportion of male and female participants in nephrology trials according trial characteristics

Trial Category	No. of Studies Included in the Model	Weight, %	Random Pooled ES for Proportion of Male Enrollment	95% CI	Random Pooled ES for Proportion of Female Enrollment	95% CI	Test for Heterogeneity Between Subgroups
AKI	69	18.6	0.67	0.63 to 0.70	0.33	0.30 to 0.37	*P* = <0.001
CKD	85	23.2	0.61	0.57 to 0.65	0.39	0.35 to 0.43	
GN	25	6.5	0.48	0.37 to 0.58	0.52	0.42 to 0.63	
HD/PD	55	14.8	0.59	0.56 to 0.61	0.41	0.39 to 0.44	
Kidney transplant	139	36.8	0.64	0.63 to 0.65	0.36	0.35 to 0.37	
Publication year							*P* = 0.84
2000–2006	91	24.1	0.61	0.59 to 0.64	0.39	0.36 to 0.41	
2007–2011	77	20.6	0.63	0.58 to 0.68	0.37	0.32 to 0.42	
2012–2016	94	25.2	0.63	0.59 to 0.67	0.37	0.33 to 0.41	
2017–2021	111	29.9	0.61	0.59 to 0.64	0.39	0.36 to 0.41	
Enrollment site							*P* = 0.02
United States only	60	15.7	0.61	0.54 to 0.67	0.39	0.33 to 0.46	
Global	82	22.6	0.61	0.59 to 0.64	0.39	0.36 to 0.41	
Europe	149	39.6	0.63	0.62 to 0.65	0.37	0.35 to 0.38	
Asia	41	10.8	0.57	0.53 to 0.61	0.43	0.39 to 0.47	
International (non-US)	41	11.0	0.64	0.62 to 0.66	0.36	0.34 to 0.38	
Intervention type							*P* = 0.83
Drug	280	75	0.67	0.53 to 0.81	0.38	0.36 to 0.40	
Device	4	1.0	0.58	0.50 to 0.65	0.42	0.35 to 0.50	
Dialysis	34	9.1	0.64	0.60 to 0.68	0.36	0.32 to 0.40	
Procedure/surgery	9	2.4	0.61	0.50 to 0.71	0.39	0.29 to 0.50	
Exercise/meditation	2	0.5	0.61	0.57 to 0.66	0.39	0.34 to 0.43	
Care delivery	36	9.7	0.61	0.55 to 0.67	0.39	0.33 to 0.45	
Other	8	2.1	0.67	0.53 to 0.81	0.33	0.19 to 0.47	

I^2^ trial categories=76%–99%, I^2^ publication year=95%–99%, I^2^ enrollment site=95%–99%. ES, estimated proportion; CI, confidence interval; HD, hemodialysis; PD, peritoneal dialysis.

**Table 4:** Meta-regression with the proportion of participants enrolled who were categorized as women as the dependent variable

Proportion of Women	Coefficient	SD	*P* Value
Publication year	−0.0034	0.02	0.87
Trial target condition	0.06	0.02	0.01
Enrollment site	0.01	0.02	0.62
Intervention - drug	−0.06	0.09	0.47
Intervention - dialysis	−0.15	0.10	0.14
Intervention - device	−0.09	0.21	0.67

*N*=373 trials. Model Akaike information criterion=0.93, Bayesian information criterion=214.

### Comparisons with Global and US Disease Prevalence

**Figure 2:**
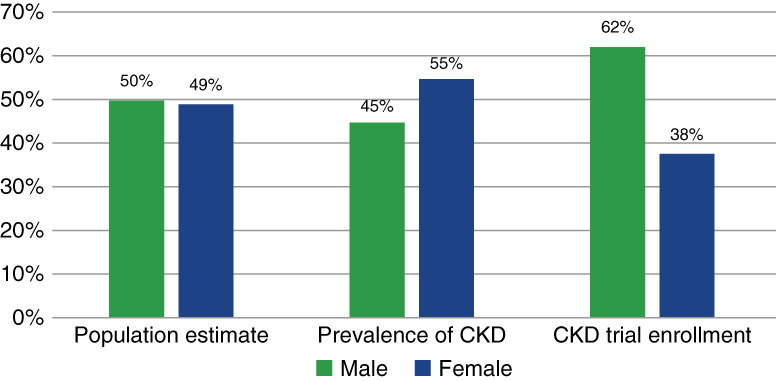
Comparison of worldwide population sex distribution, global CKD prevalence, and CKD trial enrollment for all CKD trials worldwide.

**Figure 3:**
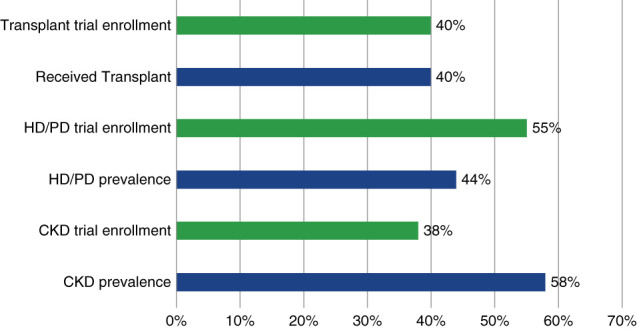
**Proportion of women enrolled in US trials compared with the proportion of female patients with the disease in the population.** Blue bars show prevalence of disease and red bars show proportion of women enrolled in trials.

Women constitute roughly half of the world's population, and CKD has been shown to be more common in women globally at 55% compared with men 45%.^6,13^ As shown in Figure 2, global enrollment of women was disproportionately low in CKD trials relative to both the proportion of women in the world population and estimates of CKD prevalence. Although women account for roughly half of the US population and for a higher proportion of patients with CKD, the proportion of women on dialysis or receiving a transplant was lower compared with men. Differences between US disease prevalence and enrollment proportion in transplant trials were negligible, whereas women were enrolled at a disproportionately high rate in dialysis trials. By contrast, the proportion of women enrolled in US CKD trials (38%) was disproportionately low relative to CKD prevalence (52%) Figure 3.

## Discussion

Underrepresentation of women in clinical trials is pervasive and reflects a history of policies and misplaced fears that have resulted in exclusion of women from trial enrollment and may negatively affect care where sex-related differences in biology affect responses to therapy.^3^ Although gender differences in the epidemiology of kidney disease are well described, the enrollment of women in kidney disease trials has not been comprehensively reported. To better understand the representation of women in nephrology trials, we performed a comprehensive review and meta-analysis of high-impact nephrology RCTs published over a 20-year period.

Our analysis has several important findings: Male participants account for most (62%) of the trial participants overall and across multiple nephrology trial categories, with the exception of GN trials in which women were enrolled at marginally higher rates than men. This trend was true throughout the world, but notably US trials tend to enroll relatively higher proportions of female participants (male to female ratio of 1.7 globally versus 1.2 in the United States). Male-dominant enrollment has been persistent without qualitative changes and has remained at a proportion at or above 60% between 2000 and 2021. Imbalances were particularly striking in AKI and transplant trials in which men accounted for approximately two of every three trial participants. Gender-related enrollment disparities were also notable in CKD trials in which women accounted for <40% participants despite accounting for more than half of the background population of patients with CKD. This pattern was similar in the United States and the rest of the world. By contrast, in trials conducted in the United States, enrollment of women in transplant trials was qualitatively in line with estimates of gender-specific disease prevalence, and women were enrolled in disproportionately higher numbers relative to ESKD prevalence in dialysis trials. Trial condition type seems to be the most important factor explaining the variation in enrollment of women.

In 1993, FDA guidelines were published that lifted previous restrictions on the participation of women with childbearing potential from entering phase I and II trials. In addition to emphasizing fair representation of women in drug trials, the guidelines identified three specific pharmacokinetic issues of concern meriting study in women, including the effect of stages of menstrual cycle, oral contraceptives, and the effect of drug on the oral contraceptives.^16^ Despite these revised guidelines, enrollment of women in trials in general has continued to lag. For instance, one recent study reported the participation to disease prevalence ratio of women enrolled in recent landmark trials published in the *New England Journal of Medicine*, examining sodium glucose transporter 2 inhibitors, glucagon-like peptide-1 receptor agonists, and nonsteroid mineralocorticoid antagonists. Women comprised a minority of the study populations with enrollment ranging from 30% to 36% of participants, with a participation to prevalence ratio ranging from 0.56 to 0.72.^17^ Our analysis is consistent with these findings while extending them further with a longer period included, beyond a single journal, to diseases other than diabetic CKD.

The stark disparities we observed in enrollment of women compared with men in nephrology trials are likely to affect generalizability of the trial findings and may contribute to differences in outcomes of kidney disease in men and women, particularly for CKD, AKI, and transplant trials in which women accounted for <40% of trial participants worldwide. Several studies have demonstrated key sex-related differences in the metabolism, handling, toxicity, and efficacy profile of commonly used drugs, often on *post hoc* analysis of original trial data.^18,19^ Indeed, heterogeneous pharmacodynamics and differences in the risk of adverse reactions between men and women^20^ may put women at a disadvantage for understanding the efficacy and safety of treatment options if women are not represented in trials. For instance, ten prescription drugs have been withdrawn from the US market since 1997, with eight of ten posing greater risks for women than men.^21^ Understanding the biologic differences in diseases and response to therapies, especially in areas where women tend to face a higher burden of disease is thus particularly crucial. In addition to the higher burden of CKD in women than men, sex differences in the expression of drug transporters and carriers in kidney have been identified that may contribute to CKD-specific, sex-related changes in pharmacokinetics of kidney-targeted drugs in men and women. For example, a preclinical study showed a higher rate of clearance of furosemide in male rats compared with female rats with important implications for dosing and toxicity.^22^

Understanding the heterogeneity in efficacy and safety profiles of therapies requires enrolling women in sufficient numbers in kidney disease trials, ideally in large enough numbers to perform sex-specific subgroup analyses that are powered adequately to identify differential beneficial or harmful effects of kidney disease therapies. Our analysis suggests that with the exception of GN trials, kidney disease trials do not achieve this goal overall, although performance is better in US transplant and dialysis trials.

The relatively higher percentage of women enrolled in the GN trials likely reflect the high proportion of lupus nephritis trials which enrolled a majority of female patients, reflecting the higher prevalence of disease in women than men (85% women).^23^ In addition, many IgA trials were conducted in Asia where the prevalence of disease is high in women—for example, the ratio of men to women in biopsy-proven IgA in Asia is 2:1 in contrast to Northern Europe and the United States where the ratio is 6:1.^24^ Indeed, the region of enrollment seemed to be important more broadly. Although there is a male predominance in nephrology trial enrollment globally, there was a distinctly different pattern in trials conducted in the United States alone in which women are enrolled in slightly higher proportions. There were marked differences, for example, in the characteristics of dialysis trials conducted globally compared with those conducted solely in the United States where women were enrolled in higher numbers than men despite a lower prevalence of ESKD. Similarly, enrollment in US transplant trials within the United States seems to match the overall prevalence of kidney transplantation in men and women.

How to increase the enrollment of women in nephrology trials is uncertain. Ensuring a diverse pool of investigators and clinical trial staff may be critical. Several studies have shown that the trials with female first or last authors have a higher proportion of female enrollment and sex-specific analysis.^25,26^ While reporting of the sex of participants was nearly universal (98%) in our analysis, universal requirements for reporting sex and gender at publication could also help improve understanding and increase awareness of important sex-specific disparities. Training of research staff and investigators in recruitment strategies, and alleviating any fears in participation could also help. Working with patient advocacy groups, community organizations, and community health care providers to raise awareness regarding trials might also encourage more equitable enrollment. While not the focus of our analysis, in addition to enrollment, overall efforts from research team and community providers should likely include efforts to ensure equal retention of men and women. Dedicated reporting on sex-based dropout and loss to follow-up rates would be helpful, and it is likely that lessons on recruitment and retention can be learned from successful studies with high or universal inclusion of women, such as the Women's Health Initiative.^27^ Strategies to enhance equity in trial enrollment are summarized in Supplementary Figure 1.

A few limitations of our study should be noted. We recorded sex as a binary variable because that is the way it has been historically recorded. In the future of clinical trials, sex and gender may be recorded as nonbinary variables. A two-step approach, where trial participants are asked their sex assigned at birth and their current gender identity might be considered because the prevalence of kidney disease is disproportionately higher in transgender individuals and differs in transgender women and transgender men.^28^ In addition, we restricted our systematic review to trials published in ten high-impact journals to focus on trials with the greatest likelihood of publishing practice-changing results. Some trials that collectively play a role in advancing the field of nephrology may have been missed with this methodology. In addition, because we did quantify treatment efficacy or compare effects of alternative therapies and focused on trials that specifically chose those most likely to be practice changing, we did not analyze enrollment trends according to trial quality. However, this may be an area worth exploring in the future.

The make-up of clinical trial enrollment should ideally mirror the prevalence of kidney disease conditions in the population. Our analysis suggests that the body of nephrology trials in the last few decades has consistently failed to meet this standard. Addressing the marked disparities in the enrollment of women and men would improve understanding of discrepancies in kidney progression in men and women and how best to address them. To generate study populations that are more reflective of the kidney disease population, substantial effort is needed to enroll women and under-represented genders in clinical trials.

## Supplementary Material

## Data Availability

Anonymized data created for the study are or will be available in a persistent repository on publication. Compiled Data. Other. Excel file.

## Supplemental Material

Supplemental Table 1. Search strategy.

Supplemental Table 2. Bias assessment of trials included.

Supplemental Figure 1. Suggested ways to improve enrollment of women participants in nephrology randomized clinical trials.
