# Supplementary material for: Gender Disparities in Nephrology Trials: A Meta-Analysis of Enrollment Trends between 2000 and 2021
Source: Kidney360. 2023 Oct 27;4(11):1545–53. doi: 10.34067/KID.0000000000000281 (PMC10695639; doi:10.34067/KID.0000000000000281)

Supplementary Document: Gender disparities in nephrology trials: A meta-analysis of enrollment trends between 2000-2021

Supplementary Table 1. Search Strategy

Supplementary Table 2.

Supplementary Figure 1: Suggested ways to improve enrollment of women participants in nephrology randomized clinical trials.

Supplementary Table 1. Search Strategy

PubMed database search using terms including (((("kidney diseases" [MH:noexp] OR "renal insufficiency " [MH:noexp] OR "acute kidney injury" [MH:noexp] OR "renal insufficiency, chronic" [MH:noexp] OR "kidney tubular necrosis, acute" [MH:noexp] OR "kidney failure, chronic" [MH:noexp] OR "glomerulonephritis" [MH:noexp] OR "anti-glomerular basement membrane disease" [MH:noexp] OR "glomerulonephritis, IGA" [MH:noexp] OR "glomerulonephritis, membranoproliferative" [MH:noexp] OR "glomerulonephritis, membranous" [MH:noexp] OR "glomerulosclerosis, focal segmental" [MH:noexp] OR "lupus nephritis" [MH:noexp] OR "renal dialysis" [MH:noexp] OR "peritoneal dialysis" [MH:noexp] OR "peritoneal dialysis, continuous ambulatory" [MH:noexp] OR "hemodiafiltration" [MH:noexp] OR "hemodialysis, home" [MH:noexp] OR "renal replacement therapy" [MH:noexp] OR "continuous renal replacement therapy" [MH:noexp] OR "hemofiltration" [MH:noexp] OR "hemoperfusion" [MH:noexp] OR "hybrid renal replacement therapy" [MH:noexp] OR "intermittent renal replacement therapy" [MH:noexp] OR "kidney transplantation" [MH:noexp] OR "kidneys, artificial " [MH:noexp] OR "kidney diseases" [tw] OR "kidney disease" [tw] OR "CKD" [tw] OR "RPGN" [tw] OR "ARF" [tw] OR "CRRT" [tw] OR "CVVH" [tw] OR "ESKD" [tw] OR "ESRD"[tw] OR "renal insufficiency " [tw] OR "renal insufficiencies " [tw] OR "kidney insufficiency" [tw] OR "kidney insufficiencies" [tw] OR "acute kidney injury" [tw] OR "AKI" [tw] OR "acute kidney injuries" [tw] OR "chronic renal insufficiency " [tw] OR "chronic renal insufficiencies" [tw] OR "kidney tubular necrosis" [tw] OR "chronic kidney failure" [tw] OR "glomerulonephritis" [tw] OR "anti-glomerular basement membrane disease" [tw] OR "glomerulosclerosis" [tw] OR "lupus nephritis" [tw] OR "kidney dialysis" [tw] OR "renal dialysis" [tw] OR "peritoneal dialysis" [tw] OR "hemodiafiltration" [tw] OR "hemodialysis" [tw] OR "renal replacement therapy" [tw] OR "kidney replacement therapy" [tw] OR "renal replacement therapies" [tw] OR "kidney replacement therapies" [tw] OR "hemofiltration" [tw] OR "hemoperfusion" [tw] OR "kidney transplantation" [tw] OR "kidney transplant" [tw] OR "kidney transplants" [tw] OR "renal transplantation" [tw] OR "renal transplant" [tw] OR "renal transplants" [tw] OR "artificial kidney" [tw] OR "artificial kidneys" [tw])) AND ("Clin J Am Soc Nephrol"[Journal] OR "J Am Soc Nephrol"[Journal] OR "Am J Kidney Dis"[Journal] OR "Nephrol Dial Transplant"[Journal] OR "Am J Transplant"[Journal] OR "Kidney Int"[Journal] OR "Lancet"[Journal] OR "N Engl J Med"[Journal] OR "JAMA"[Journal] OR "BMJ"[Journal])) AND ((((randomized controlled trial [pt] OR "controlled clinical trial"[Publication Type] OR "randomized"[Title/Abstract] OR "placebo"[Title/Abstract]) OR ("clinical trials as topic" [mesh: noexp]) OR (randomly [tiab] OR trial [ti])) NOT (animals [mh] NOT humans [mh])))) AND (2000:2030 [pdat]

Supplementary Table 2.

Bias Trials included = 380	High	Low	Unsure
Sequence generation (selection bias)	96	283	1
Allocation concealment (selection bias)	155	223	2
Blinding of participants and personnel (Performance bias)	264	114	2
Blinding of outcome assessment (detection bias)	317	62	1
Incomplete outcome data (attrition bias)	67	285	28
Selective reporting (reporting bias)	56	301	23
Other sources of bias	293	59	28

Supplementary Figure 1: Suggested ways to improve enrollment of women participants in nephrology randomized clinical trials.

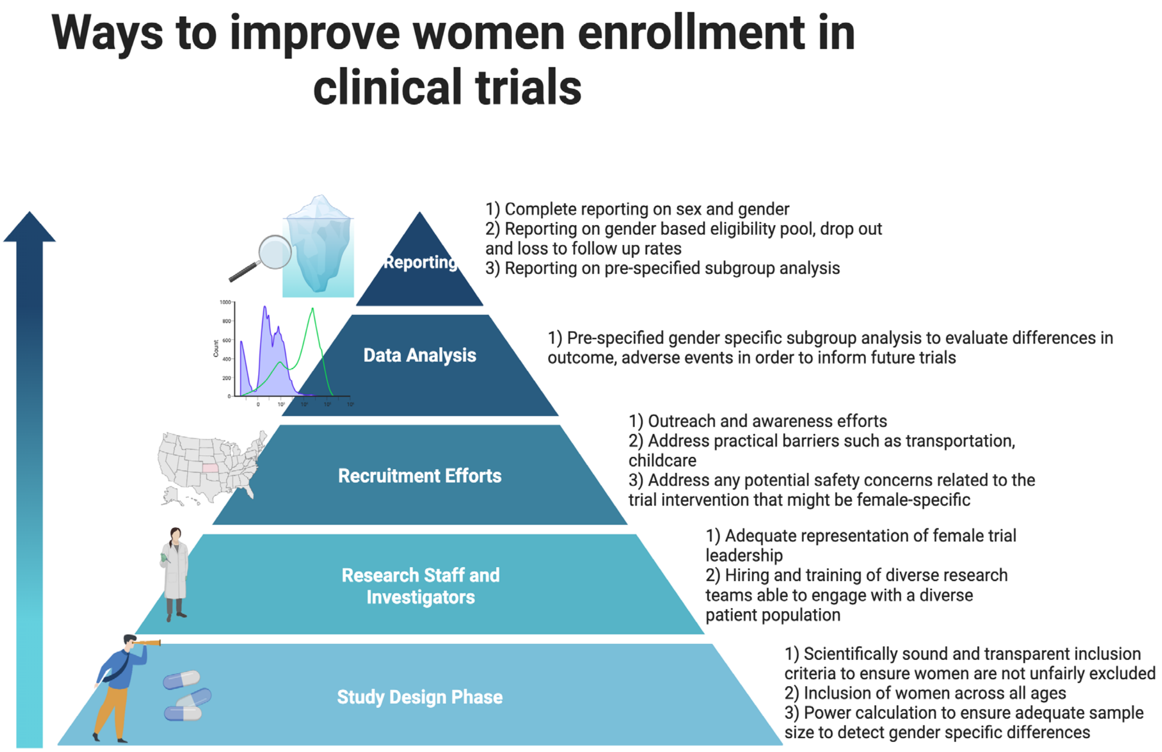

Supplement: Supplementary file 1 [file kidney360-4-1545-s001.pdf]
